# The Impact of Growth Hormone Deficiency on Endothelial Function in Childhood Brain Cancer Survivors

**DOI:** 10.3390/cancers17233746

**Published:** 2025-11-24

**Authors:** Marco Crocco, Federica Malerba, Noemi Zampatti, Valentina Nosratian, Carolina Bigatti, Marta Panciroli, Amanda Casirati, Maria Grazia Calevo, Mohamad Maghnie, Natascia Di Iorgi

**Affiliations:** 1 Department of Neuroscience, Rehabilitation, Ophthalmology, Genetics, Maternal and Child Health, University of Genoa, 16132 Genoa, Italynatasciadiiorgi@gaslini.org (N.D.I.); 2 Pediatric Gastroenterology and Endoscopy Unit, IRCCS Istituto Giannina Gaslini, 16147 Genoa, Italy; 3 UOC Pediatria e Neonatologia Imperia, IRCCS Istituto Giannina Gaslini, 18100 Imperia, Italy; 4 Allergy Center, IRCCS Istituto Giannina Gaslini, 16147 Genoa, Italy; 5 Nephrology, Dialysis and Transplantation Unit, IRCCS Istituto Giannina Gaslini, 16147 Genoa, Italy; 6 Epidemiology and Biostatistics Unit, Scientific Directorate, IRCCS Istituto Giannina Gaslini, 16147 Genoa, Italy; 7 Pediatric Endocrinology Unit, Department of Pediatrics, IRCCS Istituto Giannina Gaslini, 16147 Genoa, Italy

**Keywords:** endothelial dysfunction, childhood cancer survivors, brain tumor, growth hormone, adiponectin, lipid, EndoPAT, cardiovascular risk, late effect

## Abstract

**Simple Summary:**

Survivors of childhood brain cancer often face long-term health problems caused by cancer treatments, including endothelial damage that increases the risk of cardiovascular diseases. Growth hormone deficiency is one of the most common late effects and may worsen this risk by altering metabolism and fat distribution. This study explored how growth hormone deficiency and its treatment affect endothelial function, using a multimodal approach that combined biochemical and biophysical assessments of vascular health. We found that many survivors showed early signs of endothelial dysfunction, especially those not receiving growth hormone therapy, who also had less favorable cholesterol and adiponectin levels. Our findings suggest that monitoring and treating growth hormone deficiency could help improve cardiovascular health in this population of survivors, which remains at high risk of premature death.

**Abstract:**

**Background:** Survivors of childhood brain cancer survivors (CBCS) have a higher risk of endothelial dysfunction and cardiovascular mortality. Recombinant human growth hormone (rhGH) replacement therapy may help reduce endothelial damage and the development of cardiovascular diseases (CVD). This study aimed to assess biochemical and biophysical endothelial function in CBCS with GH deficiency (GHD). **Methods:** CBCS who were at least two years post-treatment underwent clinical evaluation, including anthropometric measurements and metabolic assessments (adiponectin, blood clotting, and lipid profile). Endothelial function was evaluated using the estimation of the reactive hyperemia index (RHI) measured by the EndoPAT 2000. A value < 1.5 was considered pathologic. CBCS without GHD served as the control group. **Results:** The study included 60 participants: 12 controls (mean age 14 ± 4.7 years) and 48 CBCS with GHD (mean age 16.6 ± 4.9 years), 8 of whom were not receiving rhGH therapy. The cohort showed a high prevalence of abnormal RHI values. Although there were no significant differences in weight or body mass index between groups, those with GHD, especially those not on rhGH therapy, had a higher prevalence of an RHI < 1.5, lower pathological adiponectin levels and a disrupted lipid profile. **Conclusions:** CBCS exhibited altered RHI values consistent with early endothelial biophysical dysfunction. Among patients with GHD, this impairment was further associated with an adverse lipid profile and signs of adipose tissue dysfunction. Recombinant growth hormone replacement therapy may contribute to a partial improvement in biochemical indicators of endothelial function.

## 1. Introduction

The number of childhood cancer survivors (CCS) has significantly increased in recent years, thanks to advancements in treatment and supportive care. Estimates suggest that there are between 450 and 1240 survivors per million people, depending on the methods and study designs used [1,2]. However, oncological treatments can lead to “late effects”, which are a range of complications that may appear months or even years after therapy [3]. In a cohort of CCS monitored for 27 years following cranial radiotherapy, at least one HP deficit was reported in 51% of patients. The hypothalamus is particularly vulnerable to radiation damage, with cranial radiotherapy being the major risk for hypothalamic-pituitary (HP) dysfunction in a dose-dependent manner [4]. Unlike dysfunction caused by tumor lesions or surgery, hormone deficiencies and radiological abnormalities, such as pituitary atrophy, can emerge decades after radiotherapy [5,6,7].

Brain tumors are the second most common neoplasm in children, representing approximately 25% of all tumors [8]. In the initial years after treatment, excess mortality in CBCS is often related to the primary tumor [9,10]. However, in long-term survivors, cardiovascular diseases (CVD) become the leading cause of premature death [11]. The cumulative cardiovascular mortality risk and the incidence of endocrine late effects do not appear to diminish over time [12,13,14]. Endocrine late effects, such as GHD, are frequently under-diagnosed due to a lack of specialized centers for the long-term follow-up [15].

The pathophysiological mechanisms behind CVD in CCS are multifactorial. Endothelial dysfunction, the earliest identifiable stage of atherosclerosis, disrupts endothelial homeostasis, contributing to CVD [16]. Various biochemical and biophysical markers have been evaluated for their ability to detect endothelial dysfunction [17]. Although coronary angiography remains the gold-standard for assessing cardiovascular health, it is impractical for screening in pediatric populations. The Endo-PAT 2000 provides a non-invasive automated means of measuring endothelial function; its reactive hyperemia index (RHI) has shown a significant correlation with flow-mediated dilation (FMD) [18].

Growth hormone deficiency is the first and most common late endocrine effect observed in CBCS, due to the somatotropic axis particularly vulnerable to radiation. Cumulative risk of 87% for GHD occurring 2.5 years post-radiotherapy in children receiving doses greater than 37.5 Gy [19]. Late endocrine effects continue to develop decades after cancer treatment, leading to delays in diagnosis and management of endocrine disorders [19,20], which further increase CVD risk [21]. Evidence indicates that GHD may contribute to endothelial dysfunction by reducing nitric oxide production, promoting unfavorable metabolic changes, and increasing inflammation, all of which can lead to vascular damage and accelerating atherosclerosis, finally increasing CVD risk [22,23,24,25]. However, research on the long-term effects of GHD on endothelial dysfunction in CBCS remains limited.

The aim is to evaluate endothelial function and in CBCS with GHD, both with or without treatment with rhGH treatment, with CBCS without GHD as controls.

## 2. Materials and Methods

### 2.1. Subjects

This cross-sectional study enrolled all childhood brain cancer survivors who were consecutively evaluated at the Endocrine and Neuro-oncology Units of the IRCCS Istituto Giannina Gaslini, Genoa, between March 2020 and April 2022. Eligibility criteria included survivors aged 5–25 years who had completed oncological treatment at least two years before study entry. Exclusion criteria comprised tumor-associated syndromes (e.g., Turner, Klinefelter, Noonan, or other chromosomal abnormalities) and congenital cardiac anomalies. The study was conducted in accordance with the Declaration of Helsinki and received approval from the Regional Ethics Committee of Liguria (protocol code 334/2019). Written informed consent was obtained from all participants and/or their legal guardians prior to enrollment.

### 2.2. Clinical, Biochemical and Biophysical Endothelial Evaluation

All patients underwent a clinical and anthropometric (height, weight, body mass index (BMI), Waist to Hip Ratio (WHR)) evaluation. Obesity was defined by a BMI ≥ 95th percentile for age and sex in children and adolescents [26] and BMI ≥ 30 in adults. Blood pressure was evaluated as the average of three measurements using a cuff appropriate to arm size. Blood pressure percentiles were calculated based on age and height [27].

Each pituitary axis—growth hormone (GH), adrenocorticotropic hormone (ACTH), thyroid-stimulating hormone (TSH), gonadotropins (LH/FSH), and prolactin—is assessed during annual follow up. According to the Endocrine Society Clinical Practice Guidelines, dynamic stimulation tests are used to confirm deficiency when indicated. For each axis, a deficiency is counted only when there is unequivocal biochemical evidence of impaired secretion, interpreted in the context of clinical symptoms and appropriate reference ranges [28]. The Italian Medicines Agency (AIFA) established a cut-off value of GH  <  8 ng/mL in two provocative tests performed on different days to diagnose GHD in children [29].

Biochemical endothelial function was assessed by measuring circulating markers of endothelial dysfunction, including components of the coagulation profile (fibrinogen, protein S, protein C, factor VIII, von Willebrand factor antigen, and homocysteine) and the lipid profile. Total cholesterol, triglycerides, and high-density lipoprotein (HDL) cholesterol were quantified using enzymatic colorimetric assays. Fasting low-density lipoprotein (LDL) cholesterol was calculated according to the Friedewald formula [30]. Reference values for plasma lipid levels were derived from the NCEP Expert Panel on Cholesterol Levels in Children [31]. Adiponectin concentrations were determined using an immunoenzymatic assay.

Biophysical endothelial function was evaluated using the Endo-PAT 2000 device (Itamar Medical Ltd., Caesarea, Israel), a non-invasive, reproducible, and operator-independent system for assessing microvascular endothelial function through peripheral arterial tonometry. The device measures changes in digital pulse volume during reactive hyperemia to calculate the reactive hyperemia index (RHI). After five minutes of brachial artery occlusion at a supra-systolic pressure (60 mmHg above systolic or up to 200 mmHg), plethysmographic biosensors record pulsatile volume changes in both fingers. The RHI is automatically computed as the ratio of post- to pre-occlusion pulse amplitude in the occluded arm, normalized to the control arm and adjusted for baseline vascular tone [32].

Bonetti et al. report a RHI of <1.35–1.49 as cut off for coronaropathy in adults [33]. In adults, an RHI cut-off of less than 1.49 predicted severe CVD with a sensitivity of 75% and a specificity of 72.9% [34]. An RHI below 1.67 suggests endothelial dysfunction with a sensitivity of 82% and a specificity of 77% [35]. There is no defined RHI cut-off value for pediatric patients [36].

### 2.3. Statistical Analysis

Descriptive statistics were generated for the whole cohort, data are described as mean and standard deviation (SD) or median and range for continuous variables, and as absolute and relative frequencies for categorical variables. Non-parametric analysis (Mann-Whitney or Kruskal-Wallis test) for continuous variables and the Chi square or Fisher’s exact test for categorical variables were used to measure differences between groups. *p* values ≤ 0.05 were considered statistically significant, and all *p* values were based on two-tailed tests. For variables with significant differences (*p* < 0.05), mean differences and corresponding 95% confidence intervals (CI) were calculated to estimate the magnitude and precision of effects. Effect sizes were determined using Cohen’s *d*, interpreted as small (0.2), medium (0.5), or large (≥0.8) [37]. Spearman’s correlation analysis was used to evaluate the relationships between different endothelial risk parameters. The relationship between IGF-1 SDS and endothelial function was first assessed through univariate linear regression analysis. Variables showing significant or near-significant associations (*p* ≤ 0.05) were subsequently included in multivariate models to adjust for potential confounders, including adiposity indices (BMI SDS, WHR), biochemical markers of metabolic syndrome (HDL cholesterol, triglycerides, insulin), cranial radiotherapy exposure, and the number of pituitary deficiencies. ROC analysis was used to find the most sensitive RHI cutoff point across the different groups. Statistical analysis was performed using SPSS version 28.0.1.0 for Windows (SPSS Inc., Chicago, IL, USA) and GraphPad Software 8 (Dotmatics, Boston, MA, USA). For this pilot study we decided to enroll all the patients evaluated for one year in our clinic.

## 3. Results

### 3.1. Clinical Characteristics

Of the 62 patients selected, 61 were found suitable for inclusion, 1 patient refused to participate, therefore 60 participants were enrolled, 32 females (53%) and 28 males (47%), mean age was 16.1 ± 4.9 years. The oncological diagnosis was: 20 sellar tumor (10 craniopharyngioma; 4 germinoma; 1 adenoma; 1 Langerhans cell histiocytosis; 4 other low grade tumor) 15 low grade glioma (9 pilocytic astrocytoma; 4 ganglioglioma; 2 pilomyxoid astrocytoma); 12 medulloblastoma; 8 other high grade tumor (3 embryonal tumor NOS; 2 pinealoblastoma; 1 germinoma; 1 anaplastic ependymoma; 1 anaplastic astrocytoma); 5 other low grade tumor (2 papilloma; 2 chordoma; 1 teratoma). The primary site of the tumor was: 30 sellar/parasellar; 10 posterior cranial fossa; 9 multiple localization; 6 hemispheric; 4 pineal gland; 1 brainstem.

Forty-eight patients were diagnosed with GHD (GHD group), of them forty are being treated with a standard dose of rhGH (rhGH group), while 8 refused or discontinued the treatment (no rhGH group); a further 12 patients had no GHD (control group). Two patients in treatment with rhGH and one patient not in treatment were taking metformin, while one patient in treatment and one not in treatment were taking a statin for hypercholesterolemia. Participant characteristics of the three groups are presented in Table 1.

Children (*n* = 24) received a rhGH dose range of 0.018–0.032 ug/kg/day (mean 5.1 ± 1.9 mg/week/m^2^ body surface) while adolescents (*n* = 16) that already reached final height a dose range of 0.2–1 mg/day (mean 1.59 ± 2 mg/week/m^2^ body). A target serum IGF-1 levels within the age-adjusted reference range was used to adjust the dose of rhGH dose during treatment, along with the growth velocity range for age and sex (and body surface area if obese). The mean duration of rhGH treatment was 49.2 ± 15.4 months.

Comparing GHD group and controls, the mean age was similar at oncological diagnosis (7.5 ± 4.5 years versus 7.0 ± 4.6 years), and at evaluation (16.6 ± 4.9 years versus 16.1 ± 4.9 years). The controls were taller than the GHD group, while there was no significant difference between the two groups for anthropometric parameters, sex, tanner stage, blood pressure, age at diagnosis and at evaluation and tumor grade. Twenty-seven (56%) of GHD patients versus 25% of control group had a tumor involving hypothalamic-pituitary region; forty (83%) GHD patients received cranial radiation (maximum mean dose on primary tumor 54.1 ± 8.4 Gy) with a significant higher prevalence compared to controls (50%, *p* < 0.05). Pituitary deficiencies were more common in the GHD group than the controls (3.1 vs. 0.5). Patients treated with rhGH had similar IGF1 values to controls, while a significant difference was detected between GHD patients not receiving rhGH and both other groups.

Obesity was highly prevalent across all three study groups, with the highest frequency observed in GHD patients not receiving treatment (75.0%, 6/8), followed by GHD patients undergoing rhGH therapy (67.5%, 27/40) and controls (58.3%, 7/12). The mean BMI was within the pathological range in both GHD cohorts. While comparative statistical analyses did not demonstrate significant differences in BMI distribution (*p* = 0.68), WHR demonstrated differences among the three groups (*p* < 0.05). Mean WHR values were higher in untreated GHD patients compared with both treated GHD patients and controls (1.08 vs. 0.88 and 0.94, respectively), revealing a clinically relevant upward trend among untreated patients, indicative of an increased central adiposity burden potentially associated with the absence of rhGH therapy. Metabolic syndrome (MS) was present in 5 patients (8.3%): 3 in treatment with rhGH, 1 with GHD but not in treatment, 1 control.

### 3.2. Biochemical and Biophysical Endothelial Evaluation

**Figure 1:**
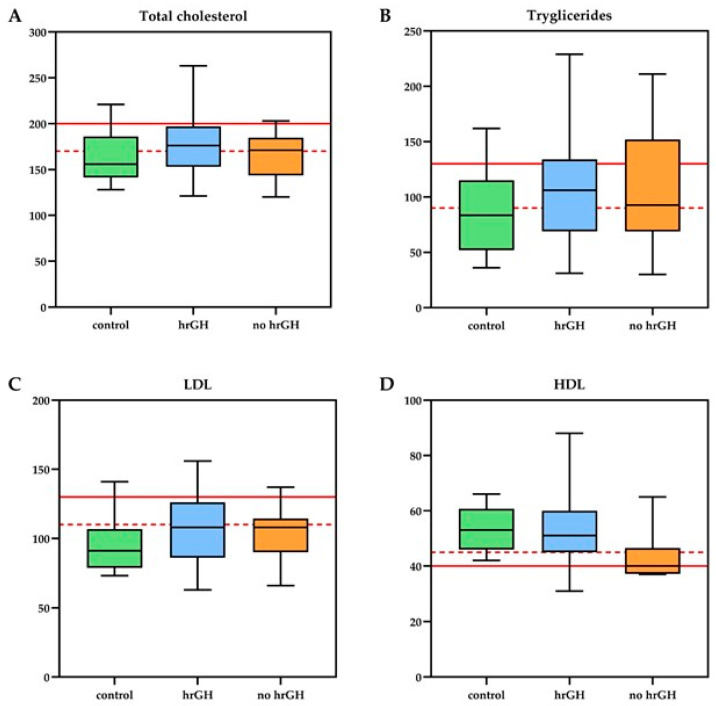
Lipid profile of Growth Hormone Deficient (GHD) patients treated with recombinant human Growth Hormone (rhGH = 40), untreated GHD patients (no rhGH = 8), and control group (*n* = 12). The panels display (**A**) Total cholesterol, (**B**) Triglycerides, (**C**) Low-Density Lipoprotein (LDL), and (**D**) High-Density Lipoprotein (HDL) levels [mg/dL]. The horizontal red lines represent the established clinical cut-off points for lipids: the dashed line indicates the threshold for acceptable/desirable levels, and the solid line denotes the threshold for pathological levels [33].

**Figure 2:**
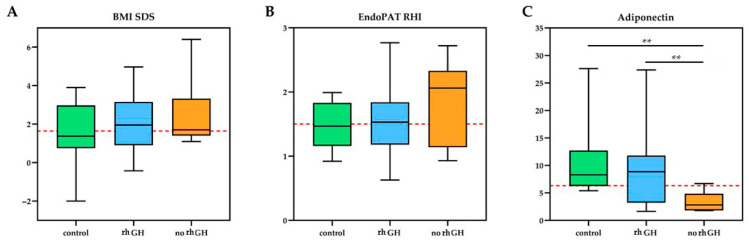
Anthropometric and endothelial function parameters in Growth Hormone Deficient (GHD) patients treated with recombinant human Growth Hormone (rhGH = 40), untreated GHD patients (no rhGH = 8), and healthy control group (*n* = 12). The box plots compare (**A**) Body Mass Index Standard Deviation Score (BMI SDS), (**B**) Reactive Hyperemia Index (RHI) measured by EndoPAT, and (**C**) Adiponectin levels across the three groups. Red lines indicate established pathological cut-off points. Statistically significant differences between the groups are denoted by the displayed *p*-values. ** *p* < 0.005.

The biochemical and biophysical endothelial function did not differ between the entire GHD group and controls. However, when considering the GHD subgroups separately, the GHD subgroups showed a significantly higher burden of vascular risk factors compared to the control group. The lipid profile showed a mean level slightly above the cut-off for total cholesterol in all three groups, with pathological triglycerides in both GHD groups and pathological HDL in patients not receiving rhGH with a significant difference: HDL concentrations were significantly higher in rhGH-treated patients compared with untreated survivors (mean difference = 0.26 mmol/L, 95% CI 0.04–0.48, *p* = 0.02, Cohen’s *d* = 0.83), while untreated patients showed lower HDL levels than controls (mean difference = −0.23 mmol/L, 95% CI −0.42 to −0.04, *p* = 0.02, d = 1.39) (Figure 1). Adiponectin levels were significantly higher in rhGH-treated than untreated subjects (mean difference = 6.2 mg/L, 95% CI 1.8–10.6, *p* = 0.007, d = 1.19) and markedly lower in the no-rhGH group compared with controls (mean difference = −7.1 mg/L, 95% CI −10.5 to −3.7, *p* < 0.001, d = 1.74) (Figure 2).

There was no significant difference between the three groups for other biochemical and biophysical endothelial parameters including EndoPAT RHI (Table 2). However, the prevalence of endothelial dysfunction evaluated by EndoPAT was high in all groups based on the cut-off reported in adults (RHI < 1.5): 3/7 (42.8%) GHD not on treatment, 18/38 (47.3%) GHD receiving rhGH and 5/9 (55.6%) controls. Of 26 patients with endothelial dysfunction evidenced by RHI, 23 (88%) received cranial radiotherapy (versus 20/28, 71%, in patients with RHI > 1.5; *p* = n.s.), 19 (73%) chemotherapies (versus 17/28, 61%; *p* = n.s.), 16 (62%) were obese (versus 20/28, 71%; *p* = n.s.), 12 (46%) had high grade (III–IV) tumors (versus 12/28, 61%; *p* = n.s.), while the mean number of pituitary deficiencies was 2.6 (versus 2.8; *p* = n.s.) with GHD in 21 (81%) (versus 24/28, 85%; *p* = n.s.). ROC analysis was performed to evaluate the discriminatory ability of the RHI among treatment groups (Figure S1). The analysis revealed limited accuracy of RHI in differentiating between patients treated with rhGH, untreated GHD patients, and controls. The comparison between rhGH and no-rhGH groups yielded the highest AUC (0.58) with an optimal cutoff of 2.1 (sensitivity = 57%, specificity = 82%), suggesting only a weak ability to distinguish treated from untreated subjects.

**Figure 3:**
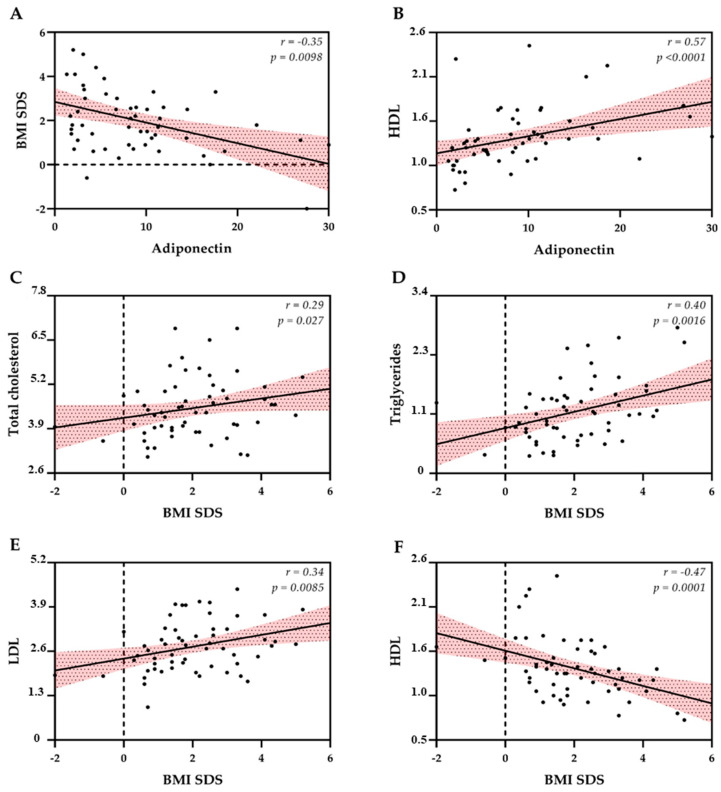
Spearman’s correlation between different endothelial risk parameters. Black lines represent linear regression fits, with shaded pink areas indicating the 95% confidence interval. The dashed lines denote BMI SDS = 0. (**A**) Negative correlation between adiponectin and BMI SDS. (**B**) Positive correlation between adiponectin and HDL cholesterol. (**C**–**F**) Correlations between BMI SDS and lipid profile: total cholesterol, triglycerides, LDL and HDL cholesterol. BMI SDS = Body Mass Index Standard Deviation Score; HDL = High-Density Lipoprotein; LDL = Low-Density Lipoprotein.

Significant correlations were found between BMI, lipid levels and adiponectin (Figure 3), while no correlations were found between RHI and the same CVD. The analysis revealed a strong positive correlation between WHR and BMI SDS *n* = 46; r = 0.71, *p* < 0.005), underscoring the convergent measurement of central and general adiposity by these metrics. Conversely, WHR exhibited a strong negative correlation with Adiponectin (*n* = 40; r = −0.68, *p* < 0.005), indicating that higher central fat accumulation is significantly associated with lower circulating levels of this protective adipokine. The remaining variables (triglycerides, total cholesterol, HDL, LDL and RHI) showed only weak or negligible correlations with WHR.

Univariate analysis showed a significant negative association between IGF-1 SDS and RHI (β = −0.03, *p* = 0.04), confirming the predictive value of IGF-1 for endothelial function. In multivariate analyses, the models demonstrated limited stability due to the small sample size; however, IGF-1 SDS remained a negative predictor of RHI after adjustment for adiposity indices (BMI SDS and WHR) and key biochemical markers of metabolic syndrome (HDL cholesterol, triglycerides, and insulin). When additional covariates, including cranial radiotherapy exposure and the number of pituitary deficiencies, were considered, the negative association between IGF-1 SDS and RHI persisted, although the overall model lost statistical robustness.

The primary complications observed with the Endo-PAT 2000 were localized and self-resolving. Paresthesia during the last part of measurement was a very common occurrence, affecting over half of the patients undergoing the examination. Notably, one patient presented with petechiae on the limb subjected to vascular occlusion at the conclusion of the examination, and another patient experienced persistent burning neurological pain for approximately 24 h post-procedure.

## 4. Discussion

In this pilot study, we found that children and adolescents survivors of brain cancer exhibit an increased risk of endothelial dysfunction after a median of seven years post-treatment. To our knowledge, this is the first study to assess both biochemical and biophysical aspects of endothelial function in CBCS and the influence of GHD and its replacement therapy on endothelial function. While there is extensive research on CVD in long-term adult survivors, data in pediatric cohorts remain limited [38,39]. By focusing on these age groups, our study adds valuable insights into the cardiovascular health of CBCS, emphasizing the multifactorial nature of endothelial dysfunction precociously detectable in a very young cohort, which may contribute to increased cardiovascular mortality in adulthood.

The high prevalence of endothelial dysfunction in our cohort may be associated with several factors, including the late effects of treatments such as chemotherapy and cranial radiotherapy, as well as subsequent pituitary deficiencies. Our findings underscore the complex interplay between cancer, treatment modalities, and their effects on lipid metabolism. Thus, a comprehensive evaluation is essential for identifying early cardiovascular risks during childhood and transitional ages. Previous research by Zelcer et al. demonstrated impaired endothelial function in 26 young Hodgkin lymphoma survivors who had received mediastinal radiation, reflected by a low RHI (mean value of 1.67 ± 0.39), similar to our findings [40]. In a smaller study of 16 acute lymphoid leukemia survivors, while traditional cardiovascular risk factors were comparable to their siblings, they exhibited poorer vascular health, as measured by RHI [41]. However, this study did not assess lipid profiles, whereas our findings indicate pathological lipid levels in both GHD groups, with better profiles in those receiving rhGH treatment. While univariate analysis confirmed a significant negative association between IGF-1 SDS and RHI, this relationship weakened in multivariate models after adjustment for adiposity indices and key biochemical markers of metabolic syndrome. Despite this reduction in statistical significance, IGF-1 SDS consistently remained a negative predictor of endothelial function, suggesting that lower IGF-1 levels may contribute to endothelial impairment in CBCS with GHD. The inclusion of additional covariates (e.g., cranial radiotherapy exposure and the number of pituitary deficiencies) did not materially change the direction of the association but further reduced model robustness, likely reflecting the small and heterogeneous sample size. Nevertheless, the persistence of a negative trend between IGF-1 SDS and RHI supports the hypothesis that IGF-1 deficiency may play an independent role in endothelial dysfunction.

The high prevalence of biophysical dysfunction, evidenced by an RHI below 1.5 across all groups, is consistent with previous reports of a substantial cardiovascular risk burden in CBCS [12]. The mean RHI in our cohort was close to the pathological threshold, with a considerable proportion of participants showing values below 1.5, indicative of severe endothelial dysfunction when compared with healthy adolescents, whose mean RHI has been reported as 1.85 ± 0.6 [42] and also when compared with optimal cutoff identified in our cohort (RHI = 2.1). A ROC analysis evaluating the capacity of RHI revealed modest accuracy. Biophysical assessment using RHI demonstrated limited discriminatory ability in our cohort to differentiate among treatment groups, with the highest AUC observed between the rhGH-treated and untreated GHD groups. This may partly explain the lack of consistent biophysical evidence of endothelial dysfunction despite biochemical abnormalities between the three groups. This discrepancy likely reflects age-related and physiological differences in vascular reactivity and endothelial responsiveness, as pediatric and adolescent populations typically exhibit higher baseline RHI values due to greater vascular compliance and nitric oxide bioavailability. Moreover, disease- and therapy-related factors—such as residual GH secretion, treatment duration, lipid profile and pubertal status—may modulate endothelial function differently than in healthy children and adolescents, leading to a wired RHI distribution. These findings highlight the need for age-specific reference values validation, and longitudinal assessments of RHI to better define clinically meaningful thresholds in CBCS populations.

In a U.S.-based registry of 11,985 long-term survivors under age 19, five-and ten-year survival rates were 74.1% and 70.7%, respectively [43]. Excess mortality among long-term survivors is primarily linked to cardiovascular and cerebrovascular diseases, often worsened by hormone deficiencies. The Childhood Cancer Survivor Study found that 18% of 1607 long-term CBCS reported one or more adverse cardiac or circulatory events. Notably, the risk of stroke as a late effect was over 40 times higher in these patients compared to their siblings, while angina-like symptoms were twice as prevalent [44]. One of the largest studies on childhood cancer survivors, with a mean follow-up of 27 years, indicated interactions between pituitary dysfunction and the impact of untreated GHD on cardiovascular health and body composition. Specifically, untreated GHD was significantly associated with reduced muscle mass and exercise tolerance. The pituitary deficits were independently linked to abdominal obesity, reduced energy expenditure, and muscle weakness [21]. Decreased lean mass, energy expenditure, and muscle strength are three of the five components of the frailty phenotype, which can be indicative of early mortality risk. In the SJLIFE cohort, the prevalence of frailty phenotype was found to be 13.1%, which is comparable to the 9.9% observed in the general population aged 65 and older [45]. Our study revealed that the prevalence of pituitary deficits, including GHD and hypogonadism, is notably higher than what was reported in the St. Jude cohort (80% versus 47% for GHD and 33% versus 11% for hypogonadism). This highlights the significant frailty within the CBCS group. Implementing hormone replacement therapies that improve body composition may contribute to sustainable improvements in cardiovascular late effects.

Recent studies convincingly show that both excess GH levels and GHD are associated with increased CVD risk [46,47]. A meta-analysis including 19,153 adults with hypopituitarism and a follow-up duration of more than 99,000 person years, demonstrated that hypopituitarism was associated with a weighted standard mortality ratio (SMR) of 1.99, with 95% confidence intervals of 1.21–2.76. Notably, rhGH replacement significantly lowered mortality in adults (SMR 1.15; 95% CI 1.05–1.24) compared to those not receiving rhGH, (SMR 2.40; 95% CI 1.46–3.34). The higher prevalence of pituitary deficits and neuromotor involvement in CBCS may explain some differences observed between CBCS and other cancer types [48].

Body composition is crucial in the metabolic health of CCS. The CCS with a higher fat-to-lean mass ratio (FLR) values demonstrate increased trunk fat, elevated triglycerides, and insulin resistance, alongside lower lean mass and IGF-1 levels-factors that collectively predispose survivors to the risk of metabolic syndrome and cardiovascular issues [49]. Changes in metabolism and body composition that begin during oncological treatment often persist long-term characterized by muscle mass loss and fat mass gain. Both BMI and WHR indicated a greater adiposity burden in GHD patients, particularly in those not receiving rhGH therapy. While BMI differences did not reach statistical significance, a clear upward trend was evident in untreated patients, consistent with previous studies reporting increased total adiposity in GHD due to impaired lipolysis and reduced energy expenditure [50,51]. WHR is a stronger predictor of cardiometabolic risk than BMI and it has a robust association with both general adiposity and key endocrine markers of metabolic dysfunction [52]. WHR is a useful marker for monitoring treatment efficacy beyond BMI alone. In our study WHR was significantly higher in the untreated group, patients on rhGH replacement demonstrated intermediate values, suggesting partial mitigation of central adiposity, in accordance with literature demonstrating that rhGH improves body composition by decreasing visceral fat while increasing lean mass, reducing the risk of sarcopenic obesity [53,54]. Clinically, these findings reinforce the importance of early initiation and long-term continuation of rhGH therapy to reduce cardiometabolic risk. Metabolic syndrome affects 8% of our cohort, with the global prevalence of metabolic syndrome in 2020 estimated, in a recent systematic review with modeling analysis, at 2.8% for children and 4.8% for adolescents [55]. These findings underscore the urgent need for early interventions focused on maintaining healthy body composition through diet and exercise, to mitigate long-term cardiovascular risks.

Adiponectin, a hormone produced by adipose tissue, plays a vital role in regulating glucose levels and fatty acid metabolism, offering protective effects against chronic low-grade inflammation [56]. Previous studies have shown that patients with adiponectin levels below 6.3 mg/L exhibit coronary endothelial dysfunction, with high specificity regarding blood flow and coronary artery diameter (85% and 88%, respectively) [57]. Low serum levels strongly correlated with endothelial dysfunction in peripheral arteries and considered an independent cardiovascular risk factor, indicating that GHD treatment may influence cardiovascular risk through yet unidentified mechanism [58]. In our study, it was expected that untreated GHD patients would have low adiponectin levels, while rhGH replacement appeared to improve these levels and reduce cardiovascular risk factors. Interestingly, despite similar body mass index and other risk factors, differences in lipid levels emerged between untreated GHD patients and other groups. This aligns with findings by Lanes et al. who showed adiponectin levels and unfavorable plasma lipid profiles in adolescents with GHD, which improved with rhGH treatment [59]. However, we did not find significant differences in biophysical endothelial function, as measured by EndoPAT 2000, between GHD groups and controls. It is worth highlighting that the mean HDL level was significantly lower in untreated patients than in those receiving rhGH; low HDL represents one of the IDF criteria for metabolic syndrome, while hypertension and glucose intolerance typically develop later in life [60,61]. Furthermore, newer targeted therapies may be associated with an increased risk of dyslipidemia necessitating thorough evaluations of cumulative risks associated with both standard and novel-treatments for optimal management of potential adverse effects [62].

As reported in our study, there is often a discrepancy between biochemical markers such as lipids and adiponectin, and biophysical EndoPAT measures of endothelial function [63]. Biochemical markers like adiponectin and lipid profiles reflect systemic metabolic and inflammatory status, and are associated with cardiovascular risk and adiposity distribution [64]. Adiponectin, for example, is inversely correlated with central adiposity and lipid parameters, but its relationship with other biochemical markers is complex and sometimes independent of visceral fat or lipid levels, i.e., genetics, diet, daily habits [65,66,67]. Studies have shown poor correlation between RHI and both biochemical markers and other biophysical measures [63,68], suggesting that RHI captures aspects of vascular function that are not reflected by circulating biomarkers. This discrepancy highlights that biochemical and biophysical measures may provide complementary but non-overlapping information about cardiovascular risk and endothelial health.

Endothelial dysfunction is a systemic vascular disorder involved in the development of atherosclerosis and is an independent predictor of cardiovascular events [69,70,71]. Early-stage atherosclerosis may be reversible, making the detection of endothelial dysfunction crucial for preventing progression and reducing CVD. Identifying patients at increased risk for CVD would enable efforts to preserve or restore endothelial function. Therapeutic approaches can be categorized into primary therapy focuses on maintaining healthy endothelial function by addressing cardiovascular risk factors through optimal lifestyle changes (e.g., diet, exercise, weight control, smoking cessation), and secondary therapy, which aims to preserve the function of already damaged endothelium and delay atherosclerosis progression by managing underlying cardiovascular risk factors and diseases [72].

The risks associated with the EndoPAT test are minimal and relate primarily to the temporary discomfort caused by the blood pressure cuff inflation; however, caution is required in special situations such as patients with thrombocytopenia, coagulopathies, and upper limb neuropathies.

This exploratory, hypothesis-generating pilot study [73] has limitations that warrant caution in generalizing the findings. Larger, multicenter studies with adequately powered multivariate designs are warranted to confirm these findings and to better delineate the relative contributions of treatment, metabolic, and endocrine factors to vascular health in this population. All participants were Caucasian and recruited from a single national reference center for endocrine late effects in CBCS, which may limit the generalizability of the results. The relatively small and heterogeneous cohort (*n* = 60) from a single center also reduces statistical power and precludes multivariate analysis. Moreover, the lack of a healthy control group represents a major limitation, as using only CBCS without GHD as controls makes it difficult to determine how endothelial function in survivors compares with the general pediatric population. Our study did not include a comparison with other biophysical measures of endothelial function. Among the currently available techniques, FMD, carotid intima–media thickness (cIMT) and PWV are the most widely recommended [74,75]. However, their use in clinical and research settings remains limited due to the need for specialized operator training, the requirement for specific ultrasonographic equipment, and the absence of standardized measurement protocols [20,29]. Additional factors, such as patients’ neuromotor status and dietary habits, may have also influenced the outcomes. The study’s inclusion criteria were well defined, and the consecutive enrollment of a brain tumor cohort with a high acceptance rate enhances the validity of the results. Nonetheless, the instability of multivariate models highlights the methodological limitations inherent to pilot studies with limited statistical power. Given the exploratory nature of this pilot study and the limited sample size, no formal correction for other multiple testing was applied. Consequently, the findings should be interpreted with caution, as the risk of type I error cannot be excluded despite the consistency and biological plausibility of the observed associations.

## 5. Conclusions

This study provides preliminary insights into the pathophysiological mechanisms contributing to cardiovascular risk in CBCS, highlighting the involvement of both biochemical and biophysical markers of endothelial dysfunction, as well as the possible influence of GHD and its treatment. Although the findings suggest a potential beneficial role of rhGH therapy on certain cardiovascular risk parameters, these results should be interpreted with caution given the limited sample size and exploratory design. The study reinforces the substantial cardiovascular risk burden faced by this population and underscores the importance of long-term monitoring of endocrine, metabolic, and endothelial health. This approach is essential for tailoring interventions that can mitigate cardiovascular risks and enhance health outcomes in this growing group of survivors.

## Figures and Tables

**Table 1:** Demographic and clinical characteristics of 60 childhood brain cancer survivors.

Characteristics	rhGH Group (*n* = 40)	No rhGH Group (*n* = 8)	CG (*n* = 12)	*p* Value rhGH vs. No rhGH	*p* Value rhGH vs. CG	*p* Value No rhGH vs. CG	Mean Difference [95% CI]; Cohen’s *d*
Age at diagnosis:							
mean years ± SD	7.6 ± 4.5	6.9 ± 5	5 ± 4.9	0.69	0.20	0.79	
Age at the evaluation:							
mean years ± SD	16.2 ± 4.8	18.2 ± 5.4	14 ± 4.7	0.33	0.15	0.11	
Time cancer diagnosis–evaluation:							
mean years ± SD	8.7 ± 4.6	11.4 ± 5.1	9 ± 4.9	0.16	0.95	0.38	
Time cancer diagnosis–end of treatments:							
mean years ± SD	7.2 ± 4.5	7.8 ± 6.4	6.9 ± 5.6	0.94	0.62	0.91	
Cranial radiotherapy:							
mean Gy ± SD	54.1 ± 8.2	54.5 ± 9.8	55.5 ± 9	0.89	0.23	0.44	
Pituitary deficiencies:							
mean numbers ± SD	**3.2 ± 1.2**	**2.9 ± 1.5**	**0.5 ± 0.5**	0.52	**0.0001**	**0.0001**	rhGH vs. CG: 2.7 [2.1–3.3]; d = 2.40 No rhGH vs. CG: 2.4 [1.5–3.3]; d = 1.95
Tanner stage:							
mean ± SD	3.5 ± 1.5	4 ± 1.6	3.1 ± 1.4	0.29	0.34	0.13	
Weight:							
mean kg ± SD	61.3 ± 22.1	66.8 ± 24.8	57.2 ± 19.6	0.56	0.66	0.34	
Height:							
mean cm ± SD	153.5 ± 16.7	156.5 ± 19.8	152.6 ± 11.8	0.78	0.75	0.85	
mean SDS ± SD	−0.9 ± 1.6	−1.1 ± 1.8	0.2 ± 1.4	0.80	0.06	0.16	
genetic target SDS ± SD	−0.4 ± 1	−0.6 ± 0.7	−0.0 ± 1	0.65	0.29	0.18	
BMI:							
mean kg/m^2^ ± SD	25.2 ± 5.6	26.5 ± 6.4	23.8 ± 5	0.60	0.38	0.21	
mean SDS ± SD	2.1 ± 1.4	2.5 ± 1.8	1.6 ± 1.7	0.75	0.51	0.21	
Waist to Hip Ratio							
mean ± SD	0.92 ± 0.2	0.90 ± 0.1	0.95 ± 0.1	0.92	0.05	0.21	rhGH vs. CG: −0.03 [−0.06–0.00]; d = 0.18
Body area surface:							
mean m^2^ ± SD	1.6 ± 0.4	1.7 ± 0.4	1.5 ± 0.3	0.56	0.47	0.27	
Fasting glucose:							
mean mmol/L ± SD	4.8 ± 0.4	4.8 ± 0.4	4.9 ± 0.8	0.82	0.35	0.49	
Fasting insulin:							
mean uU/mL	16.3 ± 8.8	16.9 ± 11.5	18.7 ± 12.1	0.94	0.71	0.78	

CG = control group; CI = confidence intervals; BMI = body mass index; Gy = grays; rhGH = recombinant human growth hormone; SDS = standard deviation score. Bold numbers indicate values showing a statistically significant difference.

**Table 2:** Biochemical and biophysical endothelial parameters of 60 childhood brain cancer survivors.

Characteristic	rhGH Group (*n* = 40)	No rhGH Group (*n* = 8)	CG (*n* = 12)	*p* Value rhGH vs. No rhGH	*p* Value rhGH vs. CG	*p* Value No rhGH vs. CG	Mean Difference [95% CI]; Cohen’s *d*
Lipid metabolism:							
triglycerides mean mmol/L ± SD	1.24 ± 0.6	1.21 ± 0.6	0.99 ± 0.4	0.86	0.23	0.62	
cholesterol mean mmol/L ± SD	4.61 ± 0.9	4.29 ± 0.7	4.25 ± 0.8	0.43	0.21	0.73	
HDL mean mmol/L ± SD	**1.39 ± 0.4**	**1.13 ± 0.2**	**1.36 ± 0.2**	**0.02**	0.89	**0.02**	rhGH vs. No rhGH: 0.26 [0.04–0.48]; d = 0.83 No rhGH vs. CG: −0.23 [−0.42–−0.04]; d = 1.39
LDL mean mmol/L ± SD	2.82 ± 0.8	2.69 ± 0.5	2.5 ± 0.6	0.73	0.17	0.30	
adiponectin mg/L ± SD	**9.6 ± 7.2**	**3.4 ± 1.8**	**10.5 ± 6.7**	**0.007**	0.72	**0.0001**	rhGH vs. No rhGH: 6.2 [1.8–10.6]; d = 1.19 No rhGH vs. CG: −7.1 [−10.5–−3.7]; d = 1.74
Prothrombotic factors:							
SBP mean Hgmm ± SD	112.6 ± 12.1	113.6 ± 14.2	105.8 ± 7.2	0.84	0.07	0.47	
DBP mean Hgmm ± SD	66.6 ± 10.2	65 ± 8.5	63.17 ± 7.00	0.67	0.23	0.85	
fibrinogen mean mg/dL ± SD	277.5 ± 50	297.4 ± 88.1	290.8 ± 72.1	0.69	0.48	0.90	
homocysteine mean umol/L ± SD	14.3 ± 21.4	19.4 ± 14.7	13.3 ± 5.8	0.10	0.09	0.36	
Endothelial function:							
EndoPAT RHI mean ± SD	1.6 ± 0.6	1.8 ± 0.7	1.4 ± 0.5	0.53	0.57	0.14	
EndoPAT ln RHI mean ± SD	0.4 ± 0.4	0.5 ± 0.4	0.3 ± 0.5	0.51	0.39	0.54	

CG = control group; DBP = diastolic blood pressure; HDL = high-density lipoprotein; LDL = low-density lipoprotein; rhGH = recombinant human growth hormone; RHI = reactive hyperemia index; SBP = systolic blood pressure; SD = standard deviation; vs. = versus. Bold numbers indicate values showing a statistically significant difference.

## Data Availability

The data presented in this study is available on request from the corresponding author.

## Supplementary Materials

The following supporting information can be downloaded at: https://www.mdpi.com/article/10.3390/cancers17233746/s1, Figure S1: ROC (Receiver Operating Characteristic) curves for the Reactive Hyperemia Index (RHI) across study groups.
