# Supplementary material for: The Impact of Growth Hormone Deficiency on Endothelial Function in Childhood Brain Cancer Survivors
Source: Cancers (Basel). 2025 Nov 24;17(23):3746. doi: 10.3390/cancers17233746 (PMC12691069; doi:10.3390/cancers17233746)
Supplement: Supplementary file 1 [file cancers-17-03746-s001.zip › cancers-3899769-supplementary.pdf]

cancers

MDPI

Figure S1. ROC (Receiver Operating Characteristic) curves for the Reactive Hyperemia Index (RHI) across study groups.

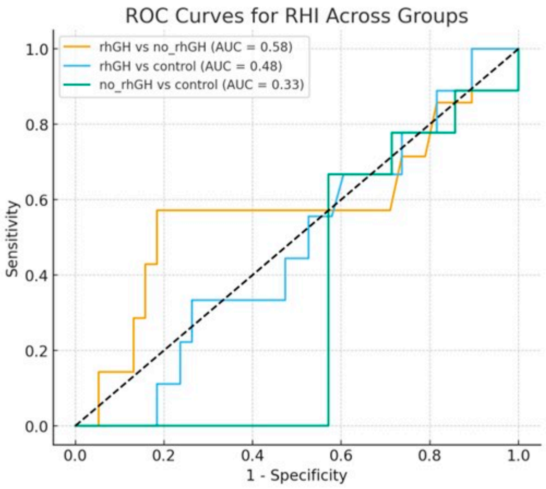

Comparison	AUC	Optimal Cut-off	Sensitivity	Specificity
rhGH vs no rhGH	0.58	2.06	0.57	0.82
rhGH vs controls	0.48	0.92	1.00	0.11
No rhGH vs controls	0.33	1.41	0.67	0.43

Figure S1. ROC (Receiver Operating Characteristic) curves for the Reactive Hyperemia Index (RHI) across study groups. The curves illustrate the discriminative ability of RHI to differentiate between subjects treated with recombinant human growth hormone (rhGH), untreated subjects (no rhGH), and healthy controls. The corresponding values for area under the curve (AUC), optimal cut-off, sensitivity, and specificity for each comparison are reported in the table below.
